# Mincing bovine articular cartilage with commercially available shavers reduces the viability of chondrocytes compared to scalpel mincing

**DOI:** 10.1186/s40634-023-00661-5

**Published:** 2023-09-28

**Authors:** Lukas B. Moser, Christoph Bauer, Alexander Otahal, Daniela Kern, Dietmar Dammerer, Thore Zantop, Stefan Nehrer

**Affiliations:** 1 Center for Regenerative Medicine, Department for Health Sciences, Medicine and Research, University for Continuing Education Krems, Dr.-Karl-Dorrek-Straße 30, 3500 Krems, Austria; 2 Department of Orthopaedics and Traumatology, University Hospital Krems, 3500 Krems, Austria; 3 Sporthopaedicum Straubing, Straubing, Germany; 4 Department for Health Sciences, Medicine and Research, University for Continuing Education Krems, 3500 Krems, Austria

**Keywords:** Cartilage, Minced cartilage, Chondrocytes, Chondrocyte viability, Cartilage defect, Scalpel mincing, Shaver mincing

## Abstract

**Purpose:**

The study aimed to compare the effect of mincing bovine articular cartilage with different shaver blades on chondrocyte viability.

**Methods:**

Bovine articular cartilage was harvested either with a scalpel or with three different shaver blades (2.5 mm, 3.5 mm, or 4.2 mm) from a commercially available shaver. The cartilage harvested with a scalpel was then minced into fragments smaller than 1 mm^3^ with a scalpel. All four conditions were cultivated in a culture medium for seven days. After Day 1 and Day 7, the following measurements were performed: metabolic activity, RNA isolation, and gene expression of anabolic (COL2A1 and ACAN) and catabolic genes (MMP1 and MMP13), live/dead staining and visualization using confocal microscopy, and flow cytometric characterization of minced cartilage chondrocytes.

**Results:**

Mincing the cartilage with shavers significantly reduced metabolic activity after one and seven days compared to scalpel mincing (*p* < 0.001). Gene expression of anabolic genes (COL2A1 and ACAN) was reduced, while catabolic genes (MMP1 and MMP13) were increased after day 7 in all shaver conditions. Confocal microscopy showed a thin line of dead cells at the lesion side with viable cells beneath for the scalpel mincing and a higher number of dead cells diffusely distributed in the shaver conditions. After seven days, there was a significant decrease in viable cells in the shaver conditions compared to scalpel mincing (*p* < 0.05). Flow cytometric characterization revealed fewer intact cells and proportionally more dead cells in all shaver conditions compared to the scalpel mincing.

**Conclusion:**

Mincing bovine articular cartilage with commercially available shavers reduces the viability of chondrocytes compared to scalpel mincing immediately after harvest and after seven days in culture. This suggests that mincing cartilage with a shaver should be considered a matrix rather than a cell therapy.

**Level of evidence:**

Level II therapeutic study.

## Introduction

Focal articular cartilage lesions typically affect young and active patients suffering from knee pain, swelling, and loss of function [21]. Those patients risk experiencing cartilage damage progression, which might lead to osteoarthritis at an early age [13]. Joint-preserving techniques aim to counteract this process. The most common methods currently used are micro-fracturing (bone marrow stimulation), osteochondral autograft/allograft transplantation, or autologous chondrocyte implantation (ACI) [26]. Studies have shown that the procedure should be selected based on the size of the cartilage defect. Microfracture should only be used in small lesions due to the increased risk of intralesional osteophytes and low-quality fibrocartilage [7]. Likewise, osteochondral autograft transplantation is recommended for only minor defects [23]. Osteochondral allograft transplantation is suitable for large osteochondral defects but is rarely used in Europe due to poor access to donor tissue [28]. ACI has been considered the gold standard for treating mid-size cartilage defects due to its favorable outcomes in randomized controlled trials [4]. Over the past decades, the ACI has steadily improved and is now in its fourth generation [6].

Nevertheless, there are also some limitations to this method [19]. It requires two different operations, which makes ACI a time-consuming procedure. Cultivation of chondrocytes takes place in the laboratory under strict conditions. Therefore, ACI is very expensive and not available everywhere in the world.

In recent years the minced cartilage procedure gained popularity, and hopes are high that this method addresses all the downsides of the established methods. In short, autologous cartilage is harvested from the edge of the defect or a non-weight-bearing region, minced into small pieces, and reimplanted into the cartilage defect in the same operation [22]. The method was first described by Albrecht et al. in a rabbit model using cartilage chips [1]. However, it was not until two decades later that further studies on animal models were published [8, 14]. Finally, the first clinical studies have been published, showing promising results in outcome scores [5, 16, 25]. Medical companies have recognized the potential of this method and are investing in standardized products such as shavers or cartilage harvesting devices.

Furthermore, they promote mixing the minced cartilage with autologous blood products. The problem is that there is still no standardization based on scientific evidence. In all published clinical studies, the mincing was performed with a scalpel, whereas recently, shaver mincing has become more popular among surgeons. However, we do not know if and how the shavers affect chondrocyte viability.

The study aimed to investigate how mincing the cartilage with commercially available shavers impairs the chondrocyte viability and whether the size of the shaver matters.

## Methods

A total of four bovine knees from slaughtered cows (age ranges from 18 to 20 months) were included in this study. The bovine knee was dissected, and the articular side of the femur was exposed. Bovine cartilage was harvested from the femoral condyles with three different sizes of a standard shaver blade used for arthroscopic cartilage debridement (Aggressive Full Radius Resector 2.5, 3.5, and 4.2 mm, KARL STORZ SE & Co. KG) collected with a commercially available filter tube (Specimen Collector – Scoop, Nico Corporation). Harvesting was performed under semi-sterile conditions in a water bath container. Approximately the same contact area of the cartilage was used to obtain a comparable quantity. The shaver settings have been standardized at 2,500 rpm in an oscillating motion. Cartilage was also obtained with a surgical scalpel and minced into small fragments (1 mm^3^). In total, four conditions were used: (i) scalpel minced, (ii) shaver minced – 2.5 mm, (iii) shaver minced – 3.5 mm, (iv) shaver minced – 4.2 mm. Samples of the conditions were incubated in six-well plates in culture medium (DMEM/F-12 supplemented with 10% FCS, 2% Pen/Strep, 2.5 µg/mL Amphotericin B, and Vitamin C). Analysis was performed on Day 1 and Day 7 per the timeline below. A change of culture medium was performed after three days. Before analysis, half of the cartilage tissue for every condition was centrifuged in a cell strainer to eliminate the remaining culture medium. After this step, the cartilage of each condition was separated into different analysis methods.

### Metabolic activity

Metabolic activity was measured from the chondrocytes within the tissue using an XTT-based ex vivo proliferation assay kit according to the manufacturer’s instructions (Cell Proliferation Kit II, Roche Diagnostics, Basel, Switzerland). In brief, from each condition, a small amount (mean value of 30 mg ± 7 mg tissue) of the cartilage pieces used for Day 1 analysis were transferred with a small spatula to Eppendorf tubes to measure the weight of the tissue. Afterward, the tissue was incubated in the XTT solution (900 µL medium, 441 µL XTT reagent, and 9 µL activation reagent) for 4 h at 37 °C with surrounding air containing 5% (v/v) CO_2_. After 4 h of incubation, the XTT solution was removed and retained. Consequently, the remaining tissue was incubated with 0.5 mL dimethyl sulfoxide (DMSO) for 1 h at room temperature under continual agitation to extract the remaining tetrazolium product from the tissue. XTT and DMSO solutions were pooled, and the absorbance was measured at 492 nm and 690 nm (background wavelength) in triplicates in a 96-well plate using a multimode microplate reader (Synergy 2, Winooski, VT, USA) with Gen 5 software. Absorbance was normalized to the weight of the tissue.

### RNA isolation

For RNA isolation, cartilage was transferred with a small spatula (same procedure as metabolic activity) from each experimental condition into tubes containing MagNA Lyser Green Beads (Roche Diagnostics, Basel, Switzerland) and 300 µL of lysis buffer (10 µL β-mercaptoethanol + 290 µL RLT [from Fibrous Tissue Kit, Qiagen, Hilden, Germany]). The tubes were frozen in liquid nitrogen and placed into the MagNA Lyser (Roche Diagnostics, Basel, Switzerland) to homogenize the cartilage tissue at 6,500 rpm for 20 s. Subsequently, the tubes were allowed to cool for 2 min, and this homogenization step was repeated four times.

Each sample was then incubated with 20 µL of proteinase K (from Fibrous Tissue Kit) for 30 min to maximize RNA yield. Next, following the manufacturer’s instructions, the RNA was isolated from the tissue and eluted in 30 µL, and then stored at − 80 °C until cDNA synthesis.

### Gene expression analysis

Gene expression analysis was carried out as previously published [2]. In brief, cDNA synthesis was performed using Transcriptor First Strand cDNA Synthesis Kit (Roche, Basel, Switzerland). RNA from bacteriophage MS2 was added to stabilize the isolated RNA during cDNA synthesis. Real-time quantitative polymerase chain reaction (RT-qPCR) was performed in triplicate using the LightCycler 96 from Roche (Basel, Switzerland). In total, five genes were analyzed: collagen type 1 (COL1), collagen type 2 (COL2A1), aggrecan (ACAN), matrix metalloproteinase-1 (MMP1), and matrix metalloproteinase-13 (MMP13)—were analyzed. In addition, glyceraldehyde-3-phosphate dehydrogenase (GAPDH) was used for normalization.

### Live/dead staining (Confocal Microscopy)

Cartilage samples, including minced and shaved tissues, were transferred into a 12-well plate and washed with Dulbecco’s phosphate-buffered saline (DPBS) to remove the culture medium. The staining solution, composed of 1 µM Ethidium homodimer-1 and 1 µM Calcein AM in DPBS, was added to each well, and the plate was incubated at 37 °C for 1 h. Following incubation, the cartilage pieces were washed three times with DPBS, placed on a glass slide, mounted using one drop of ProLongTM antifade reagent as a mounting medium, and covered with a cover glass. Tissue sections were imaged using a confocal microscope (Leica TCS SP8 MP, Leica Microsystems, Wetzlar, Germany) to visualize and quantify the live and dead cells within the tissue.

In order to quantify the living and dead cells in the acquired images, they were separated into two color channels and analyzed using the ImageJ software. An area of 0.01–0.10 square inches was used to detect the cells, with a circularity value ranging from 0.05 to 1.00. The resulting counts were assigned to their respective conditions (living/dead), and the percentage distribution was calculated.

### Flow cytometric characterization of minced cartilage chondrocytes

Cell isolation from shavered or scalpel minced cartilage fragments was performed overnight via enzymatic release using Liberase™ (0.2 WU/mL, Roche Diagnostics GmbH, Mannheim, Germany) in a shaker at 30 rpm at 37 °C, as previously described [17]. On the second day, 1*10^5^ cells were resuspended in 100 µl annexin staining buffer (10 mM CaCl_2_, HEPES) and stained with 5 µl PE-labeled annexin V and 1 µl 7-Aminoactinomycin (7-AAD) from an apoptosis detection kit (BD Pharmingen, #559793). Then, the suspension was incubated for 30 min in the dark at room temperature. After 400 µl binding buffer was added, fluorescence was detected on a Cytoflex S flow cytometer. Percentages of healthy, apoptotic, and damaged cells were determined using FlowJo version 10.0.7 software. Manually minced cartilage served as control.

### Statistics

All statistical analysis was performed using GraphPad Prism Software (Version 9.3.1, GraphPad Prism Software Inc., San Diego, CA, USA). We employed a one-way ANOVA, followed by the non-parametric Kruskal–Wallis test for multiple comparisons, and further clarified with Dunn’s post hoc test. Data concerning metabolic activity, gene expression, and the percentage of viable cells are illustrated in box plots. These plots highlight the median, first and third quartiles; error bars indicate the maximum and minimum values. Flow cytometry data are presented as stacked bars. A significance threshold was established at *p* < 0.05. Significance was assessed between Day 1 and Day 7, as well as among individual groups. In the figures, statistical significance is designated as: * for *p* < 0.05, ** for *p* < 0.01, and *** for *p* < 0.001. Absence of an asterisk denotes results that are not significant.

## Results

### Cartilage shavering decreases metabolic activity of chondrocytes

**Fig. 1:**
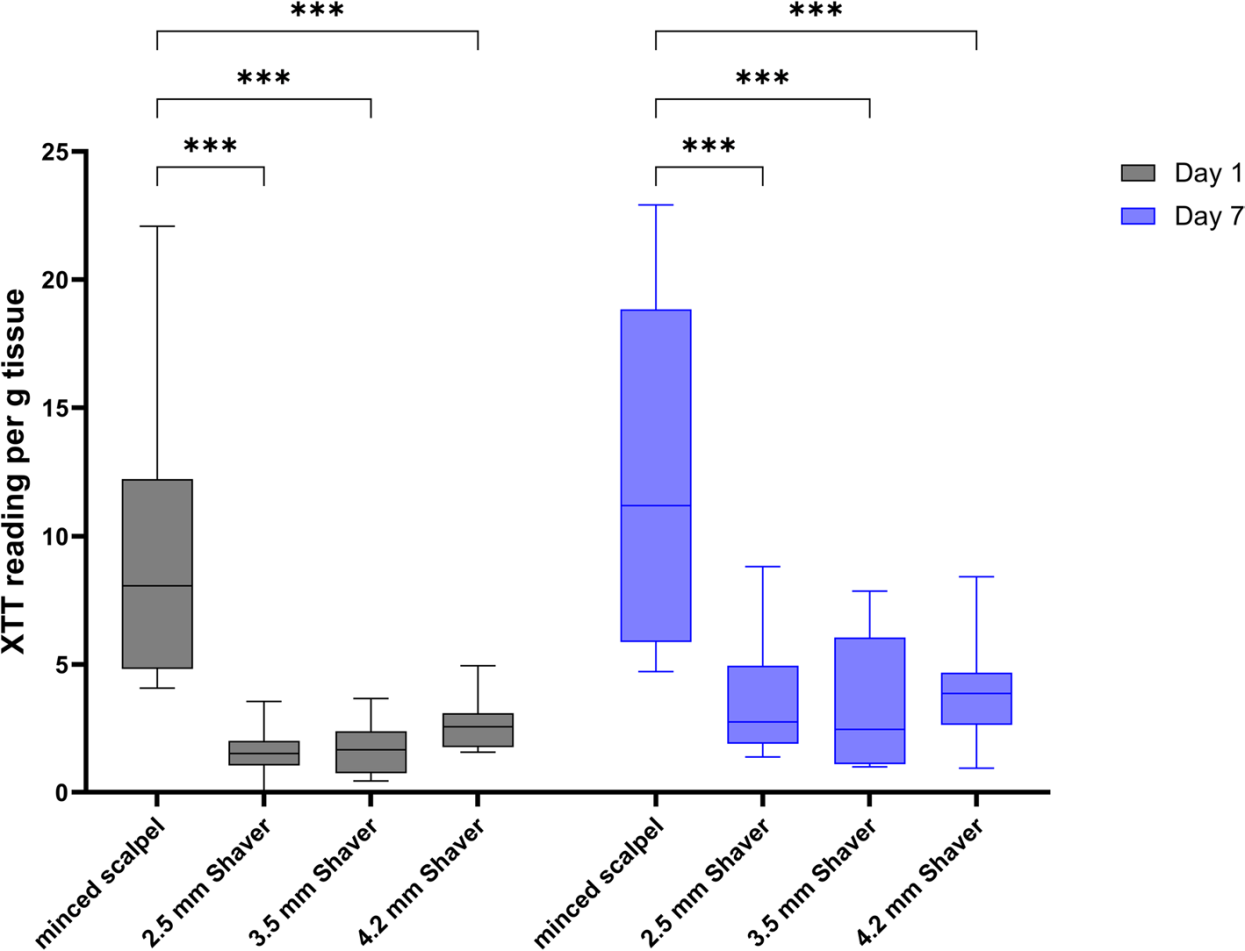
Metabolic activity (XTT reading) of the different conditions on Day 1 and Day 7 per g tissue. ***p < 0.001

Chondrocytes respond to mechanical perturbations, such as cutting or grinding, which can influence their metabolic activity or gene expression. Our results show that using shavers significantly reduced chondrocytes’ metabolic activity, independent of shaver size, compared to manually minced control (Fig. 1). This fact was shown on Day 1 and Day 7 after mincing/shaving. Additionally, we observed a slight increase in metabolic activity after seven days in all conditions.

### Cartilage shavering reduces chondrotypic gene expression

**Fig. 2:**
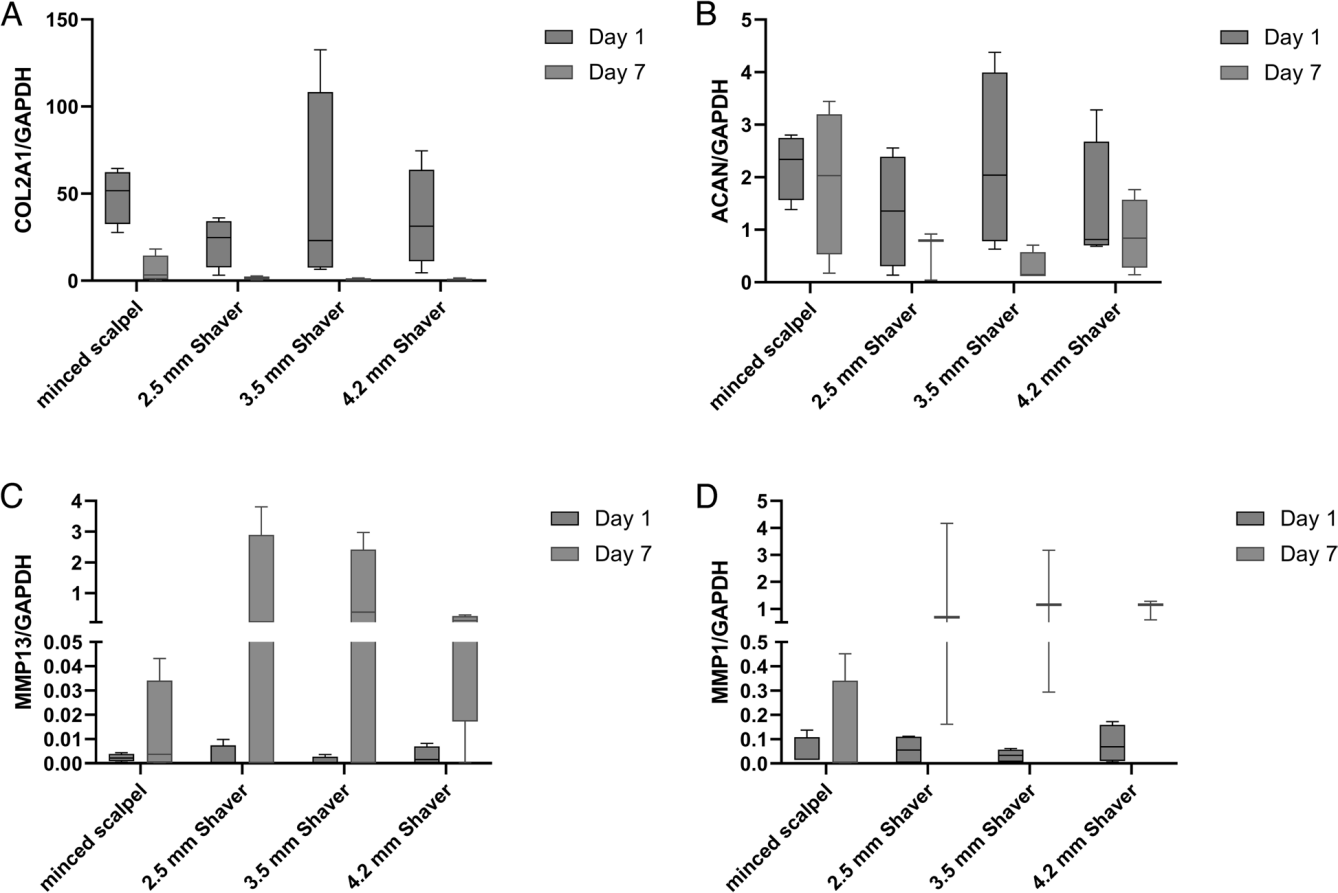
Expression of anabolic (COL2A1, ACAN) and catabolic (MMP13, MMP1) genes one day and seven days after mincing and shaving the cartilage. No significant (n.s.) differences between the different groups

In addition to its effect on metabolic activity, mechanical disturbances can alter gene expression. Our results showed that the increased cellular stress caused by using a shaver resulted in a higher reduction of COL2A1 expression (Fig. 2A) after one day compared to cutting the cartilage with a scalpel. Furthermore, the reduction in expression persisted in each group over seven days, with the COL2A1 expression in the shaver groups showing a substantial reduction.

After one day, the chondrocyte’s ACAN expression (Fig. 2B) was reduced in all minced cartilage samples. However, it significantly decreased in the 2.5 mm and 4.2 mm Shaver conditions. After seven days, the scalpel group demonstrated a similar level of gene expression to the control group, while the groups that used a shaver showed a further decline in ACAN expression.

**Fig. 3:**
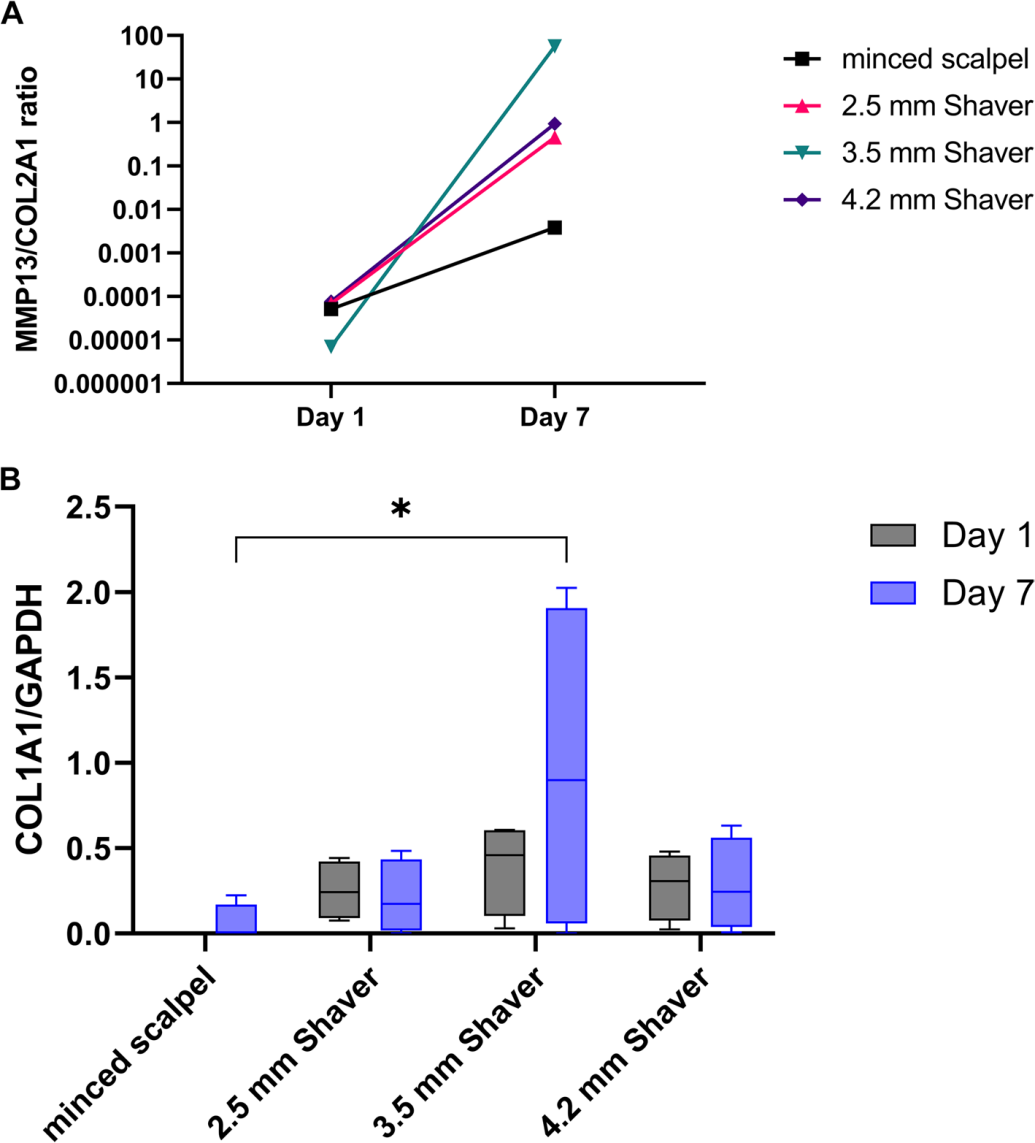
**A** Ratio of MMP13 to COL2A1 indicates an imbalance of anabolic and catabolic gene expression. **B** Expression of the gene COL1A1 on Day 1 and Day 7 after mincing and shaving the cartilage. *p < 0.05

The expression of genes encoding for two degradative enzymes, MMP13 and MMP1, was low across all conditions on Day 1 after mincing or shaving. However, after seven days, the expression of both MMPs increased in all conditions (Fig. 2C and D). Notably, the expression levels of the shaving groups were tendentially higher than that of the minced scalpel group. Furthermore, the ratio of MMP13 to COL2A1 expression (Fig. 3A) significantly increased in the shaver groups after one week of incubation, whereas the increase observed in the minced scalpel group was less prominent. The resulting values demonstrated a 100-fold or higher difference between the shaver and minced scalpel groups.

Figure 3B shows that shaving the cartilage leads to a higher COL1A1 gene expression on Day 1 than mincing the cartilage using a scalpel. After seven days, the COL1A1 expression was significantly increased for the 3.5 mm shaver group compared to the minced scalpel group. However, all other shaver conditions expressed COL1A1 at nearly the same level as on Day 1.

### Flow cytometry reveals increased chondrocyte apoptosis induced by cartilage shavering

**Fig. 4:**
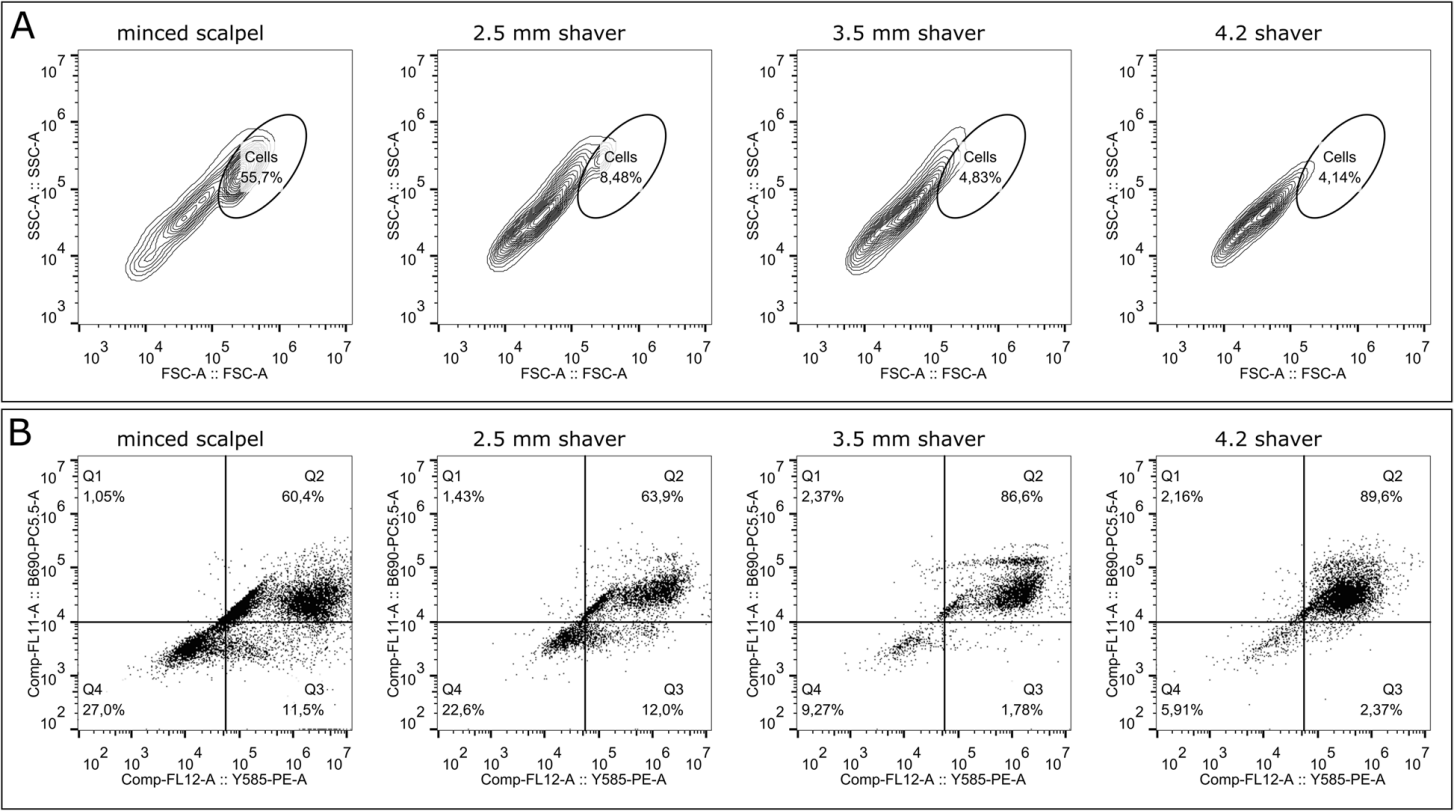
Representative flow cytometry measurements for the minced scalpel group and the three shaver conditions using annexin-V/7-AAD staining. A: Total events with a chosen cell population and remaining cell fragments. B: The lower left quadrant (Annexin-V -/7-AAD -) shows viable cells. The lower right quadrant (Annexin-V + /7-AAD -) represents early apoptotic cells. The upper left quadrant (Annexin-V -/7-AAD +) shows necrotic cells, while the right upper quadrant (Annexin-V + /7-AAD +) shows late apoptotic cells

Analysis of single cell chondrocyte suspension obtained via enzymatic digestion of cartilage fragments via flow cytometry indicated increased proportions of apoptotic and/or damaged cells. Mincing the cartilage with a shaver reduced the number of intact cells, as shown in Fig. 4A. Furthermore, all shaver conditions resulted in appearance of late apoptotic cells, as shown in Fig. 4B. Using a scalpel to mince the cartilage also led to considerably levels of primarily dead cells, but the absolute number of viable cells was substantially higher than in the shaver conditions. Specifically, there were more than 50% intact cells using a scalpel, whereas the shaver conditions resulted in less than 10% intact cells.

**Fig. 5:**
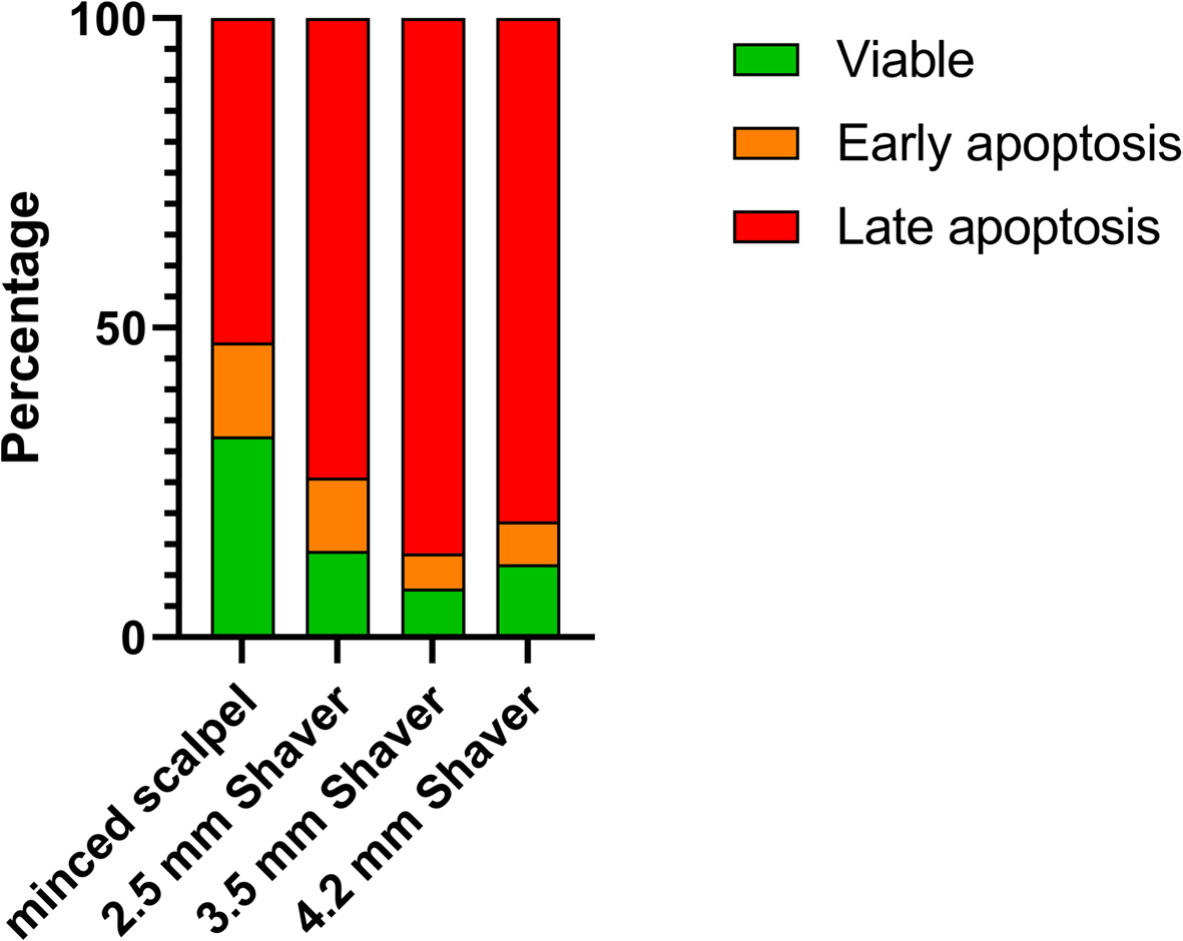
Percentage distribution of viable, early apoptotic (Annexin V) and late apoptotic cells (7-ADD)

Figure 5 depicts the mean distribution of each cell population (live, early apoptosis, and late apoptosis) among the different conditions. In the minced scalpel group, more live cells (32.4%) were observed, whereas cell viability decreased in the shaver groups (< 10%). In addition, more than 85% of cells in the shaver groups were in an apoptotic state, while this value was lower at 67% (15.1% early and 52.2% late apoptosis) in the minced scalpel group.

### Confocal microscopy confirms the detrimental effect of cartilage shavering

**Fig. 6:**
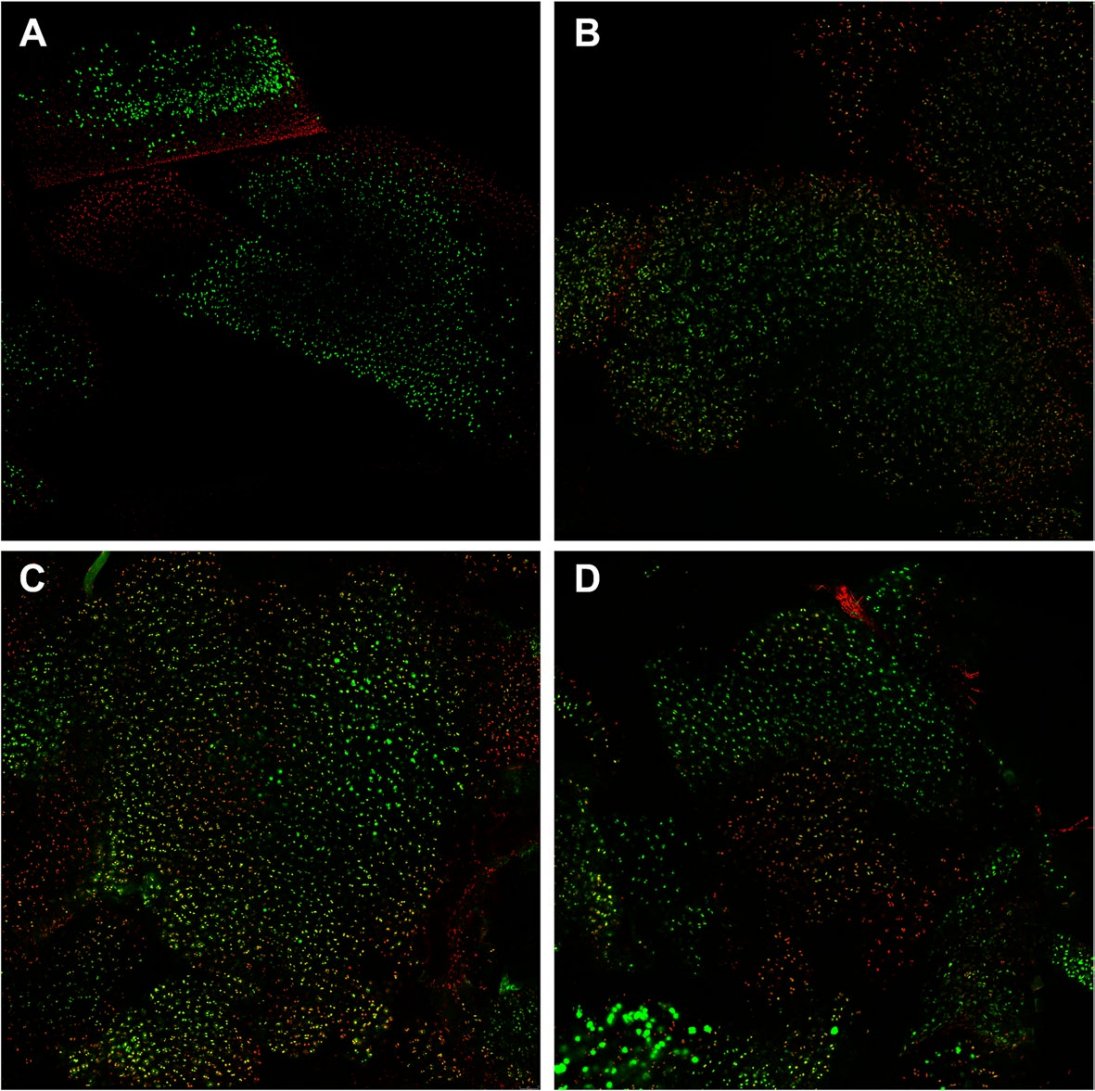
Representative confocal microscope image on Day 7. **A** minced scalpel, **B** 2.5 mm shaver, **C** 3.5 mm shaver, **D** 4.2 mm shaver. Viable cells are stained with Calcein AM (green) and dead cells with Ethidiumhomodimer-1 (red)

**Fig. 7:**
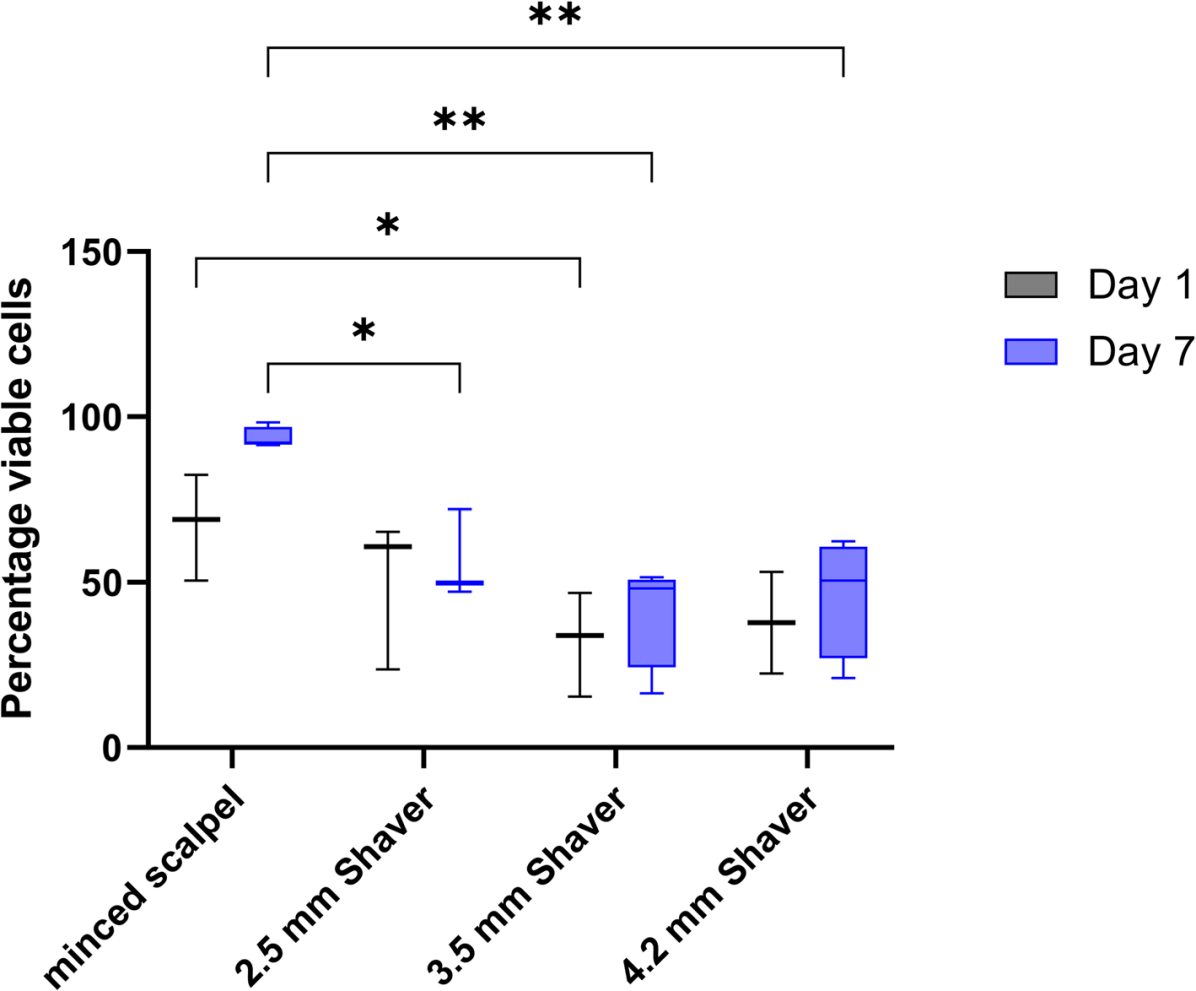
Percentage of viable cells in all conditions after Day 1 and Day 7. Values were analyzed from the confocal microscope images of the live/dead staining. *p < 0.05 and **p < 0.01

To rule out that the observed increase in Annexin V positive events determined via flow cytometry was influenced by the enzymatic release of single chondrocytes, cartilage fragments were labeled with a live/dead stain kit. The analyzed fragments from the different shaver sizes exhibited a significant decrease in viable cells in all groups after seven days compared to the minced scalpel group. While the minced scalpel group retained the original structure of the cartilage pieces, the structure was not discernible in all the shaver groups, and the fragments appeared more like slush (Fig. 6A to D). The analyzed confocal images presented in Fig. 7 reveal that approximately two-thirds of the stained chondrocytes remained viable after one day of mincing, with dead cells primarily located on the periphery of the cartilage fragments. Although the viability of the cells was reduced in all shaver groups, the decrease was not statistically significant, with approximately 50% of the cells remaining viable. After seven days of incubation, the cell viability of the minced scalpel group was around 90% based on the analyzed images.

## Discussion

The most important finding of the present study is that mincing cartilage with a shaver affects the viability of cartilage cells more than mincing cartilage with a scalpel. Redman et al. investigated the cellular responses of articular cartilage to sharp (scalpel) and blunt (trephine) trauma [20] in 2004. After ten days of incubation, cell death was assessed by labeling live/dead cells, followed by confocal microscopy investigations. In blunt trauma, a band of cell death was observed at the lesion side while a sharp trauma lead to restricted cell death. The present investigated the effect of disrupting the cartilage with shavers. The live/dead Assay analysis using confocal microscopy demonstrated the presence of multiple dead cells that were uniformly dispersed in the cartilage paste, where no distinct tissue structure was discernible. The disruptive forces that arose from using a shaver resulted in a higher incidence of cell death, particularly when the samples were subjected to a one-week incubation period.

Levinson et al. compared the chondrocyte viability and chondrocyte migration into gels (fibrin or collagen) between scalpel mincing and a mincing device [10] using cartilage from 12 patients undergoing total knee arthroplasty. After incubation of seven days, all groups had comparable high viability without any statistically significant differences (> 74%). This finding contrasts the present study’s findings on chondrocyte viability (scalpel mincing), as flow cytometry analysis detected that only half of the cells were intact after one day. The percentage of viable cells in all shaver conditions was even more dramatic, as the shaving process led to a further reduction of intact cells due to mechanical disruption. The results of the present study do not indicate a difference among the different sizes of the shaver blades (2.5 mm, 3.5 mm, 4.2 mm). Important to note here is that Levinson used a different viability assay for detecting chondrocyte viability than in the present study.

The optimal size of cartilage fragmentation is a matter of debate, and its impact on ECM production has been investigated by Bonasia et al. on cartilage derived from total hip arthroplasty [3]. The authors minced the cartilage with a scalpel into four different sizes and found out that after a cultivation for six weeks the “cartilage paste” (< 3 mm) had a significantly better ECM production per cell and a significantly better Bern score than the other groups. Although the paste is made with a scalpel, it is similar in size and consistency to mincing with a shaver. However, we could not confirm that creating a cartilage paste with a shaver yields the best results. Mincing the cartilage with a shaver decreased metabolic activity compared to manual mincing, indicating a less cell population and a possible change in the inflammatory microenvironment, which can lead to cartilage degeneration [27]. Possible cartilage degeneration processes were also shown in gene expression analysis, as anabolic genes (COL2A1 and ACAN) were reduced after one and seven days, and catabolic genes (MMP1 and MMP13) were increased. This results in the activation of chondrocytes and the generation of matrix fragments, which leads to the activation of signaling pathways, such as increased production of cytokines, chemokines, and proteolytic enzymes [12]. These factors reduce biosynthetic activity and lead to the expression of matrix-degrading enzymes, further destroying the tissue [9]. Here, the ratio of MMP13 to COL2A1, which measures the balance between anabolic and catabolic expression, underlines the predominantly catabolic effect of the shavers after seven days compared to day 1, indicating a shift towards cartilage degradation. The mechanical disruption of cartilage has been shown to impact the expression of the COL1A1 gene, which is primarily essential in bone, skin, and tendons, and is typically expressed at low levels in chondrocytes. However, tissue damage leads to an attempt to repair the tissue, often resulting in the formation of collagen type 1-rich fibrocartilage and not hyaline cartilage. This altered expression of COL1A1 has also been confirmed in a separate study dealing with cartilage disruption [18].

More than 20 years after the rabbit model by Albrecht at. some other animal studies have investigated the effect of minced cartilage in small (mouse [14] and rabbit [15]) and large (goat [11] and horse [8]) animal models showing promising results with a hyaline-like repair tissue. A total of four clinical studies on minced cartilage have been published so far. Cole et al. published 2011 a prospective clinical safety trial with a 2-year follow-up with a product that incorporated cartilage chips in a scaffold ((Cartilage Autograft Implantation System [CAIS]; DePuy Mitek, Raynham, MA). Even if the method was proven safe, the product is currently unavailable for clinical use. Three other clinical studies have shown promising results with improved clinical and radiological (MOCART) scores [5, 16, 25]. The clinical relevance of the present study becomes clear when the clinical studies are looked in detail. All studies had different approaches: Christensen et al. minced the cartilage using surgical scissors and embedded the fragments within fibrin glue [5]. Massen et al. minced the cartilage with a scalpel, embedded the fragments within fibrin glue, and covered the defect with a collagen membrane secured by sutures [16]. Wodzig et al. minced the cartilage with a scalpel and sealed the defect with fibrin glue [25]. None of the studies used a shaver that is currently available on the market, but the mincing process is most widely performed by shavers. Regardless of the mincing technique, this procedure aims to cut vital cartilage into small pieces and induce the reparative formation of new cartilage by migration, proliferation, and differentiation of chondrocytes in the targeted defect zone [22]. However, this requires sufficient viable chondrocytes not damaged by the mincing process.

The study has a considerable number of limitations. First, this study was performed on bovine cartilage, which differs from human articular cartilage. However, cartilage material from patients undergoing arthroplasty would not be sufficient since the minced cartilage procedure is supposed to be performed with healthy cartilage. Second, this study was in vitro, making it difficult to transfer the findings to physiological conditions. The present study did not perform 3D cultures, which more closely resemble a physiological environment. Also, mechanical loading was not performed, which could have affected the chondrogenesis of minced cartilage [24]. Third, only four bovine specimens were used for this study which is certainly a limited number. Fourth, only short-term effects (until D 7) were observed in the present study. A longer cultivation time would have been interesting to study time-dependent changes in more detail. However, in the present study, the comparison of Day 7 and Day 1 already showed precise results in all observations.

## Conclusion

Mincing bovine articular cartilage with commercially available shavers reduces the viability of chondrocytes compared to scalpel mincing immediately after harvest and after seven days in culture. This suggests that mincing cartilage with a shaver should be considered a matrix rather than a cell therapy. Additional standardized experimental and clinical studies investigating the mincing process with a shaver are urgently needed.

## Data Availability

The datasets used and/or analysed during the current study are available from the corresponding author on reasonable request.

## References

[1] Albrecht FH (1983). Closure of joint cartilage defects using cartilage fragments and fibrin glue. Fortschr Med.

[2] Bauer C, Niculescu-Morzsa E, Nehrer S (2017). A protocol for gene expression analysis of chondrocytes from bovine osteochondral plugs used for biotribological applications. MethodsX.

[3] Bonasia DE, Marmotti A, Mattia S, Cosentino A, Spolaore S, Governale G, Castoldi F, Rossi R (2015). The degree of chondral fragmentation affects extracellular matrix production in cartilage autograft implantation: an in vitro study. Arthroscopy.

[4] Brittberg M, Lindahl A, Nilsson A, Ohlsson C, Isaksson O, Peterson L (1994). Treatment of deep cartilage defects in the knee with autologous chondrocyte transplantation. N Engl J Med.

[5] Christensen BB, Foldager CB, Jensen J, Lind M (2015). Autologous dual-tissue transplantation for osteochondral repair: early clinical and radiological results. Cartilage.

[6] Davies RL, Kuiper NJ (2019). Regenerative medicine: a review of the evolution of Autologous Chondrocyte Implantation (ACI) therapy. Bioengineering.

[7] Erggelet C, Vavken P (2016). Microfracture for the treatment of cartilage defects in the knee joint – A golden standard?. J Clin Orthop Trauma.

[8] Frisbie DD, Lu Y, Kawcak CE, DiCarlo EF, Binette F, McIlwraith CW (2009). In vivo evaluation of autologous cartilage fragment-loaded scaffolds implanted into equine articular defects and compared with autologous chondrocyte implantation. Am J Sports Med.

[9] Kurz B, Lemke AK, Fay J, Pufe T, Grodzinsky AJ, Schünke M (2005). Pathomechanisms of cartilage destruction by mechanical injury. Ann Anat.

[10] Levinson C, Cavalli E, Sindi DM, Kessel B, Zenobi-Wong M, Preiss S, Salzmann G, Neidenbach P (2019). Chondrocytes from device-minced articular cartilage show potent outgrowth into fibrin and collagen hydrogels. Orthop J Sports Med.

[11] Lind M, Larsen A (2008). Equal cartilage repair response between autologous chondrocytes in a collagen scaffold and minced cartilage under a collagen scaffold: an in vivo study in goats. Connect Tissue Res.

[12] Loeser RF (2006). Molecular mechanisms of cartilage destruction: mechanics, inflammatory mediators, and aging collide. Arthritis Rheum.

[13] Lohmander LS, Felson D (2004). Can we identify a “high risk” patient profile to determine who will experience rapid progression of osteoarthritis?. Osteoarthritis Cartilage.

[14] Lu Y, Dhanaraj S, Wang Z, Bradley DM, Bowman SM, Cole BJ, Binette F (2006). Minced cartilage without cell culture serves as an effective intraoperative cell source for cartilage repair. J Orthop Res.

[15] Marmotti A, Bruzzone M, Bonasia DE, Castoldi F, Rossi R, Piras L, Maiello A, Realmuto C, Peretti GM (2012). One-step osteochondral repair with cartilage fragments in a composite scaffold. Knee Surg Sports Traumatol Arthrosc.

[16] Massen FK, Inauen CR, Harder LP, Runer A, Preiss S, Salzmann GM (2019). One-step autologous minced cartilage procedure for the treatment of knee joint chondral and osteochondral lesions: a series of 27 patients with 2-year follow-up. Orthop J Sports Med.

[17] Moser LB, Bauer C, Jeyakumar V, Niculescu-Morzsa E-P, Nehrer S (2021). Hyaluronic acid as a carrier supports the effects of glucocorticoids and diminishes the cytotoxic effects of local anesthetics in human articular chondrocytes in vitro. Int J Mol Sci.

[18] Natoli RM, Scott CC, Athanasiou KA (2008). Temporal effects of impact on articular cartilage cell death, gene expression, matrix biochemistry, and biomechanics. Ann Biomed Eng.

[19] Niemeyer P, Albrecht D, Andereya S, Angele P, Ateschrang A, Aurich M, Baumann M, Bosch U, Erggelet C, Fickert S, Gebhard H, Gelse K, Günther D, Hoburg A, Kasten P, Kolombe T, Madry H, Marlovits S, Meenen NM, Müller PE, Nöth U, Petersen JP, Pietschmann M, Richter W, Rolauffs B, Rhunau K, Schewe B, Steinert A, Steinwachs MR, Welsch GH, Zinser W, Fritz J (2016). Autologous chondrocyte implantation (ACI) for cartilage defects of the knee: a guideline by the working group “Clinical Tissue Regeneration” of the German Society of Orthopaedics and Trauma (DGOU). Knee.

[20] Redman SN, Dowthwaite GP, Thomson BM, Archer CW (2004). The cellular responses of articular cartilage to sharp and blunt trauma. Osteoarthritis Cartilage.

[21] Salzmann GM, Niemeyer P, Hochrein A, Stoddart MJ, Angele P (2018). Articular cartilage repair of the knee in children and adolescents. Orthop J Sports Med.

[22] Salzmann GM, Ossendorff R, Gilat R, Cole BJ (2021). Autologous minced cartilage implantation for treatment of chondral and osteochondral lesions in the knee joint: an overview. Cartilage.

[23] Sherman SL, Thyssen E, Nuelle CW (2017). Osteochondral autologous transplantation. Clin Sports Med.

[24] Wang N, Grad S, Stoddart MJ, Niemeyer P, Reising K, Schmal H, Südkamp NP, Alini M, Salzmann GM (2014). Particulate cartilage under bioreactor-induced compression and shear. Int Orthop.

[25] Wodzig MHH, Peters MJM, Emanuel KS, Van Hugten PPW, Wijnen W, Jutten LM, Boymans TA, Loeffen DV, Emans PJ (2022). Minced autologous chondral fragments with fibrin glue as a simple promising one-step cartilage repair procedure: a clinical and MRI Study at 12-month follow-up. Cartilage.

[26] York PJ, Wydra FB, Belton ME, Vidal AF (2017). Joint preservation techniques in orthopaedic surgery. Sports Health.

[27] Zheng L, Zhang Z, Sheng P, Mobasheri A (2021). The role of metabolism in chondrocyte dysfunction and the progression of osteoarthritis. Ageing Res Rev.

[28] Zouzias IC, Bugbee WD (2016). Osteochondral allograft transplantation in the Knee. Sports Med Arthrosc Rev.

